# Blood cell-derived transplants at 40

**DOI:** 10.1038/s41409-026-02865-6

**Published:** 2026-04-14

**Authors:** Anthony D. Ho, Peter Dreger, Christopher Juttner, Robert Peter Gale

**Affiliations:** 1 Department of Medicine V, University of Heidelberg, Heidelberg, Germany; 2 Past Director, National Stem Cell Foundation, Caulfield North, VIC Australia; 3 Centre for Haematology, Imperial College of Science, Technology and Medicine, London, UK

**Keywords:** Stem-cell research, Haematopoietic stem cells

## Abstract

February, 2026 is the 40th anniversary of the 1st publication of rapid recovery of bone marrow function after high-dose chemotherapy and infusion of an autologous blood- rather than a bone-marrow-derived graft. The recipient, a man with advanced Burkitt lymphoma, is in remission and alive. In the next 40 years transplants of blood-derived progenitor cells have become the most common graft for auto- and allotransplants. The rapid hematopoietic recovery after blood cell-derived grafts is associated with fewer short-term complications and better outcomes in many settings compared with bone marrow-derived grafts but may be associated with more graft-*versus*-host disease (G*v*HD) after allotransplants. Using blood-derived grafts also reduced costs, enabled the development of cell-based immune therapy and in vitro manipulation of blood-derived cells for gene and chimeric antigen receptor (CAR)-T-cell therapies.

## Introduction

February, 2026 is the 40th anniversary of the publication of rapid recovery of bone marrow function after high-dose chemo- and radiation therapy and infusion of a blood cell-derived rather than bone marrow-derived autograft [1]. The recipient was a 38-year-old man with advanced Burkitt lymphoma and proved several points which subsequently advanced the field of hematopoietic cell transplants. 1st, numbers of hematopoietic progenitor cells in blood, reflected by in vitro colony assays, can be substantially increased in the rebound phase from chemotherapy for the underlying disease. 2nd, these cells can be collected by apheresis and frozen for a future transplant. 3rd, infusing these cells after intensive chemotherapy and/or high-dose ionizing radiation results in rapid recovery of bone marrow function. Although parts of the above observations were described before the 1986 report, success of this therapy strategy was then unique. The recipient, now 78 years old, has been in remission since then and alive.

Initially met with skepticism, blood-derived progenitor cells are the most commonly used graft source for auto- and allotransplants [2]. The resulting rapid hematopoietic reconstitution is associated with fewer short-term complications and better outcomes in many, but not all settings compared with bone marrow-derived grafts. Using blood-derived grafts reduced costs and expanded access. This approach enabled transplants of specific cell types like T-cell subsets, development of chimeric antigen receptor (CAR)-T-cell therapy and genetic engineering of grafts to treat disorders like sickle cell disease (SSD) and severe combined immune disease (SCID).

## Foundations for hematopoietic progenitor cell transplants

### Stem cells as the ancestors of the cellular elements

**Fig. 1:**
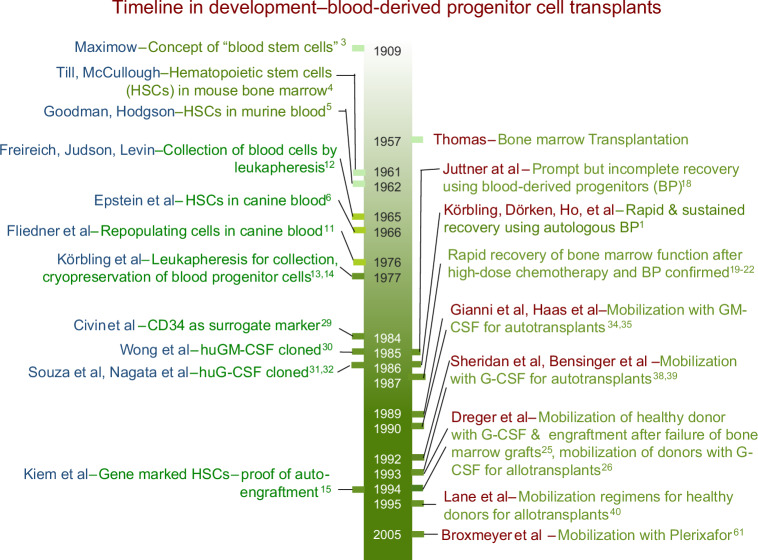
Time line in development – blood-derived progenitor cell transplants. Left side: Conceptual Foundations; right side: Enabling Technologies and Initial Reports.

The concept of *stem cells* as the ancestor of blood cells was first suggested by Maximow in 1909 (Fig. 1) [3]. He postulated “lymphocytes” (as perceived and defined in the early 1900s) were the parent of all blood cells during embryonic development and in fetal liver of mammals. It took 50 years before hematopoietic stem cells (HSC) could be proven in mouse bone marrow and spleen and subsequently defined as cells able to self-renew and differentiate into multi-lineage mature cells [4].

### Hematopoietic reconstitution using blood-derived cells

In 1962, Goodman and Hodgson reported blood-derived leukocytes from F_1_ hybrid mice promoted survival of lethally-irradiated parenteral and syngeneic mice [5]. Donor cells were identified in recipient bone marrow, blood, spleen and lymph nodes. Achieving this effect needed donor blood from 200 mice for each experiment implying this would not be a practical graft source in humans.

### Cells with repopulating capacity in mammalian blood and bone marrow

In 1966, Epstein et al. reported cross blood circulation between inbred dogs resulted in bone marrow recovery in the irradiated member of the pair [6]. Storb et al. reported that autologous blood cells could restore bone marrow function after high-dose ionizing radiation; thoracic duct cells did not [7]. In the 1970s experiments in irradiated dogs by Fliedner et al. indicated re-populating cells in shielded bone marrow restored normal bone marrow function [8, 9]. The authors concluded: (A) in steady-state there are cells with re-populating potential in blood seemingly in equilibrium with the extravascular sites; (B) there is a reserve of hematopoietic progenitor cells which can be expanded by specific stimuli like partial body irradiation and chemotherapy; (C) blood stem cells in mammalian embryos are initially in blood islands derived from hemangioblasts and subsequently establish hematopoiesis in the mesonephros, fetal liver and the bone marrow (the reason for this migration to the bone marrow is hypothesized to be for radiation protection [10]; and (D) empty spaces in the irradiated bone marrow can be repopulated by blood-derived cells indicating homing and seeding capacities [11].

In the mid-1960s to 1970s several groups developed continuous flow centrifuges which facilitated collecting large numbers of blood-derived progenitor cells in dogs, monkeys and humans [12–14]. Several research groups used genetically-marked dog blood-derived cells to show long-term persistence of infused hematopoietic cells post-infusion following high-dose radiation suggesting blood-derived progenitor cells can contribute to long-term hematopoietic recovery [15, 16].

## Early attempts of blood-derived progenitor cell transplants

Goldman et al. postulated collecting autologous WBCs from people with chronic myeloid leukemia at diagnosis might be able to restore chronic phase after these persons had transformed to blast phase [17]. There were mixed results. In 1985 Juttner *et al*. reported blood-derived progenitor cells collected soon after induction chemotherapy for acute myeloid leukemia (AML) and infused after high-dose drugs and radiation resulted in prompt but incomplete recovery of hematological parameters possibly because of too few progenitor cells [18]. In 1986 Körbling et al. reported rapid, complete recovery of bone marrow function after a blood-derived progenitor autotransplant for Burkitt lymphoma [1]. There followed several other reports of using blood-derived cells after high-dose chemotherapy and/or radiation tackling issues like mobilization of progenitor cells from the bone marrow into blood, optimizing leukapheresis techniques cell freezing and quality controls [19–22].

Use of blood-derived progenitor cells graft offers several advantages over bone marrow-derived cells including rapid recovery of bone marrow function, ability to obtain donor cells without general anesthesia, greater donor safety and less inconvenience and cost [23].

In 1993, Dreger et al. reported the successful allotransplant of blood-derived progenitor cells after the repeated failure of bone marrow grafts to recover bone marrow function in the recipient. The blood-derived graft was from the same person as the failed bone marrow-derived grafts, suggesting the superior hematopoietic potential of blood-derived progenitor cells also in allografting [24]. These authors described giving granulocyte colony stimulating factor (G-CSF) to healthy donors in 1994 [25]. Shortly after, several groups deliberated on the rapid and sustained hematologic recovery after allotransplants using blood-derived progenitors without an appreciably greater incidence of acute GVHD than would be expected with marrow [26–28].

The main challenge to using blood-derived progenitor cell grafts for transplants is the infrequency of progenitor cells in blood compared with bone marrow in steady-state. Numbers of colony forming units (CFUs) increase substantially during recovery from intensive chemotherapy and/or high-dose ionizing radiation[18–22]. Several aphereses were needed to obtain sufficient numbers of these cells.

In early 1980s CD34, a surrogate marker for cells with hematopoietic re-populating activity was identified [29]. CD34 has replaced colony-forming assays surrogate marker hematopoietic progenitor cells. Whether there is a threshold dose of CD34-positive cells and whether CD34-positive cell dose correlates with likelihood and rate of bone marrow recovery is discussed below.

## Development of hematopoietic growth factors

In the mid-1980s granulocyte-macrophage- and granulocyte-colony stimulating factors (GM-CSF, G-CSF) were molecularly cloned and became available for clinical use [30–32]. The 1st use of growth factor (GM-CSF) in human was in 1986 [33]. In 1989, Gianni et al. reported giving GM-CSF after cyclophosphamide increased numbers of blood CFU-GM which were infused into 7 subjects with cancer after total body radiation and melphalan [34]. Combined autologous blood- and bone marrow-derived cells resulted in more rapid hematologic recovery after high-dose chemotherapy compared with bone marrow-derived cells only. Others reported rapid bone marrow recovery after infusing blood-derived grafts alone obtained after administering chemotherapy and GM-CSF [35, 36].

In 1988 large numbers of blood progenitor cells were reported after using G-CSF to accelerate hematopoietic recovery after chemotherapy [37]. In 1992, Sheridan et al. reported administering G-CSF to mobilize progenitor cells after chemotherapy and using them as a graft after high-dose chemotherapy in 14 subjects [38]. Beginning in 1993 there were reports of infusing autologous blood-derived progenitor cells collected after giving G-CSF to accelerate hematopoietic recovery after chemotherapy [39].

Dreger et al. described sufficient numbers of allogeneic blood CD34-positive cells for a graft could be collected by 1-2 leukapheresis procedures [25]. In 1994, Lane et al. compared the efficacy of G-CSF, GM-CSF, versus a combination of both to mobilize CD34-positive cells into the blood from healthy donors [40]. G-CSF only, or with GM-CSF, mobilized similar numbers of progenitor cells. GM-CSF alone was less effective whilst GM-CSF followed by G-CSF was the most effective [40].

## Dose-intensity and autotransplants

Beginning in the early 1960s Skipper et al. reported the cure of mouse leukemias with increasingly high doses of drugs that, unavoidably, caused death from bone marrow failure [41]. In 1984, Hryniuk and Bush suggested the concept of dose-intensity applies to women with metastatic breast cancer [42]. These investigators and colleagues developed formulae for “received” and “relative” dose-intensity enabling analyses of actual *versus* planned dosimetry [43, 44]. These observations led to the potential strategy of collecting, freezing and re-infusing autologous bone marrow cells after high-dose chemotherapy and/or radiation and was tested in several hematologic and solid cancers. Between 1989 and 1995 there was a shift from bone-marrow-derived to blood-cell-derived grafts [45]. In one study of 19,291 autotransplants, the 100-day therapy-related mortality (TRM) decreased from 22% in 1990 to 5% in 1995. This better safety index led to widespread acceptance of blood-derived grafts. Efficacy of both types of grafts seems similar [2].

High-dose chemotherapy and/or radiation followed by a blood-derived autotransplant is safe and effective therapy for some hematologic malignancies and sometime better compared with conventional therapy [46–50]. In contrast, results of this strategy in solid cancers are mixed. For example, data from the European Society for Blood and Marrow Transplant (EBMT) indicated a benefit in non-seminomatous germ-cell tumors [51]. For breast cancer, randomized trials failed to show a convincing benefit [52, 53].

## Challenges to mobilizing blood-derived progenitor cell

Getting sufficient numbers of blood-derived progenitor cells for a infusion/transplant is challenging [54, 55]. There is a correlation between the concentration of CD34-positive cells in blood at steady state and the ability to collect adequate numbers of progenitor cells by 1-3 leukaphereses [56]. Retrospective analyses of subjects receiving induction chemotherapy and G-CSF mobilization followed by an autotransplant reported co-variates of poor-mobilizers, especially low pre-leukapheresis CD34-positive cell concentration [57]. This study also indicated that as long as > or =2.0 × 10^6^ of CD34+ cells/kg body weight have been collected, recovery of bone marrow function is rapid. Recent articles have discussed whether there is a threshold dose of CD34-positive cells for rapid bone marrow recovery and how numbers of relevant cells should be quantified [58, 59].

Human hematopoietic stem and progenitor cells are thought to adhere to the bone marrow niche by interactions between SDF1α and CXCR4 [60]. G-CSF mobilizes progenitor cells from the marrow niche by secretion of neutrophil-associated extra-cellular proteases that release stem cells from their bone marrow niche. In 2005, plerixafor, an inhibitor of the SDF1α-CXCR4 axis, was reported to increase mobilization of blood-derived progenitor cells [61, 62]. It was debatable whether higher numbers of blood-derived CD34-positive cells in a graft correlates with increased post-infusion or transplant efficacy. There is, however, no evidence there is a threshold dose of CD34-psoitive cells to achieve rapid, sustained bone marrow recovery or that more cells are more effective [57–59].

## Bone marrow *versus* blood cell-derived allotransplants

In people with a hematologic cancer and a HLA-matched related donor, a meta-analysis of randomized trials reported blood cell-derived grafts might improve disease-free survival in subjects at high-risk of relapse but at the cost of more chronic G*v*HD [63]. In a large randomized trial Anasetti et al. reported blood cell-derived grafts from HLA-matched unrelated donors had similar outcomes to bone marrow grafts [64]. Although blood-derived allografts might reduce the risk of graft-failure bone marrow grafts might diminish the risk of chronic GvHD [64]. Recent studies report blood cell-derived grafts have similar graft-failure rates, transplant-related mortality (TRM) and survival compared with bone marrow grafts. [65, 66]. A recent meta-analysis of studies of adults with aplastic anemia reported risk of G*v*HD was not increased by using blood cell-derived *versus* bone marrow grafts but this conclusion is controversial [67]. In children with hematologic cancers higher rates of chronic G*v*HD and TRM with blood cell-derived grafts favor use of bone marrow grafts [68].

There is controversy whether the higher incidence of chronic GvHD after blood cell-derived grafts is associated with fewer relapses. Some studies report less relapse and better leukemia-free survival (LFS) with blood cell-derived grafts in subjects with high-risk acute myeloid leukemia (AML) but meta-analyses of large randomized trials report only small or no significant differences in relapse risk or survival [63–65].

Reducing numbers of T-cells in blood cell-derived grafts might decrease the risk of chronic G*v*HD. Efficacy of anti-thymocyte globulin (ATG) in this context is controversial (Review: [69]. Recently, encouraging data are reported using selective naïve T-cell depletion or higher dose of ATG without increasing relapse risk or TRM [70, 71]. More data are needed.

## Blood-derived progenitor transplants and CAR-T-cells

Chimeric antigen receptor (CAR)-T-cell therapy is conceptually and technically built on experience of autotransplants using blood-derived grafts [72]. In hematological malignancies, graft-versus-host disease has been reported to correlate with disease eradication (graft-versus-leukemia) [73]. This observation laid the foundation for developing engineered autologous T-cells against tumor antigens [72]. CAR-T-cells, like blood-derived progenitor cells, are procedurally generated from T-cells obtained by leukapheresis, transduced in vitro and infused to specifically target cancer cells. This strategy is safe and effective in advanced B-cell malignancies, and has been considered as part of standard treatment algorithms [74–76].

## Conclusions

The success of blood-derived progenitor cell transplants comes from research done over decades and contributed to by many research groups globally. In the past 40 years there has been exponential growth in numbers of auto- and allotransplants using blood-derived progenitor cells. Previous advances have become standard, others discarded. Knowledge gained in developing infusions/transplants of blood-derived progenitor cells have had important consequences. What will the next 40 years bring?
